# Stability and relative validity of the Neuromuscular Disease Impact Profile (NMDIP)

**DOI:** 10.1186/s12883-017-0866-6

**Published:** 2017-05-11

**Authors:** Isaäc Bos, Jan B.M. Kuks, Josué Almansa, Hubertus P.H. Kremer, Klaske Wynia

**Affiliations:** 1 Department of Neurology, University Medical Center Groningen, University of Groningen, P.O. Box 30001, 9700 RB Groningen, The Netherlands; 2 Department of Health Sciences, Community and Occupational Health, University Medical Center Groningen, University of Groningen, Groningen, the Netherlands

**Keywords:** Neuromuscular Disease Impact Profile, Relative validity, Test-retest reliability, Stability, Criterion validity

## Abstract

**Background:**

The aim of this study was to examine the stability and relative validity (RV) of the Neuromuscular Disease Impact Profile (NMDIP) using criterion-related groups. In a previous study the NMDIP-scales showed good internal consistency, convergent and discriminant validity. Known-groups analysis showed that the NMDIP discriminates between categories of extent of limitations.

**Methods:**

A cross-sectional postal survey study was performed on patients diagnosed with a NMD and registered at the Department of Neurology, University Medical Center Groningen, the Netherlands.

Participants were asked to complete the preliminary NMDIP, the Medical Outcome study Short Form Questionnaire (SF-36), the World Health Organization Quality Of Life-abbreviation version (WHOQOL-bref), and two generic domain specific measures: the Groningen Activity Restriction Scale (GARS) and the Impact on Participation and Autonomy Questionnaire (IPAQ). The variables ‘Extent of Limitations’ and ‘Quality of Life’ were used to create criterion-related groups. Stability over time was tested using the Wilcoxon Signed Rank Test for paired samples and the intraclass correlation coefficients for repeated measures. RV was examined by comparing the ability of NMDIP with generic multidimensional health impact measures, and domain specific measures in discriminating between criterion-related subgroups using the Kruskal-Wallis H-test.

**Results:**

Response rate was 70% (*n* = 702). The NMDIP-scales showed sufficient stability over time, and satisfactory or strong RV. In general, the NMDIP scales performed as well as or better than the concurrent measurement instruments.

**Conclusions:**

The NMDIP proved to be a valid and reliable disease-targeted measure with a broad scope on physical, psychological and social functioning.

## Background

Neuromuscular Diseases (NMDs) may be caused by an abnormality of the anterior horn cells, sensory ganglion cells (neuronopathy), the peripheral nerves (neuropathy), neuromuscular junctions (myasthenia), or muscle (myopathy). Common symptoms and signs of NMD include muscle weakness, impairment in muscle endurance, involuntary muscle activity (stiffness, myotonia, cramps, and fasciculations), sensory loss, autonomic dysfunction and impairment in control of voluntary movements. Sensations of pain and fatigue are common consequences of muscle and nerve pathology [1, 2]. Easy to apply NMD-specific reliable and validated self-report assessment tools are essential for obtaining insight into the prevalence and severity of the broad range of patient perceived health-related problems in NMDs. This is important for research and for clinical practise as well, in order to narrow the gap between the clinician’s and patient’s view on the actual health situation and to help to tailor care plans to the patient’s need and preferences [3]. We therefore developed the disability-severity Neuromuscular Disease Impact Profile (NMDIP) based on the ICF-Core set for NMDs, a set of categories selected from the International Classification of Functioning, Disability and Health (ICF) [2, 4].

The NMDIP consists of 36 items that cover all ICF-components and are divided into eight scales and four single items. The NMDIP-scales showed moderate to good Cronbach’s alpha and mean inter-item correlation coefficients. Convergent and discriminant validity analysis indicated that the NMDIP measures the impact of neuromuscular disease on physical, psychological and social functioning. The NMDIP discriminates between groups of patients who differ in ‘Extent of limitations’. The four single items represent the Environmental Factors component (three items) and one Body Functions item (Seeing function) [4].

The objective of this study was to further examine the psychometric properties of the NMDIP and to build on previous studies on this measurement instrument [2, 4]. We examined its stability over time by assessing the test-retest reliability of the NMDIP-scales. We furthermore compared the ability of the NMDIP scales to discriminate between criterion related subgroups with this ability of four established concurrent measurement instruments, by assessing the Relative Validity (RV) [5, 6]. The RV coefficient indicates how much more or less valid each outcome measure is related to the best outcome measure.

## Method

### Sample and procedure

A cross sectional study, using a postal survey, was administered to patients diagnosed with a NMD who were registered at the Department of Neurology of the University Medical Center Groningen, University of Groningen, the Netherlands. Inclusion criteria for this study were: diagnosis with a NMD and representing one of Rowland’s NMD classification groups: motor-neuron disorders, muscle disorders, junction disorders and peripheral nerve disorders [7]; being aged 18 or older; being able to read and write in Dutch; and being able to give informed consent. No exclusion criteria were formulated.

A total of 1003 eligible patients were selected from the hospital patient records system. To avoid inappropriately sending the questionnaires, we crosschecked for deceased patients using the national population register. Patients received information about the study and were invited to participate.

Respondents completed demographic and disease specific questions, the NMDIP, two criterion variables to measure the ‘Extent of Limitations’ and ‘Quality of Life’. Also, concurrent measures were completed: two generic multidimensional health impact measures (the Medical Outcome study Short Form Questionnaire (SF-36) [8], and the World Health Organization Quality Of Life-abbreviation version (WHOQOL-bref) [9], and two generic domain specific measures the Groningen Activity Restriction Scale (GARS) [10] and the Impact on Participation and Autonomy Questionnaire (IPAQ) [11]. To assess stability over time, the NMDIP was administered on two occasions to patients who agreed to fill in the questionnaire twice. We, arbitrary, selected a time frame from eight to 10 weeks to be sure that patients could not remember their answers on the first questionnaire, and the likelihood of changes in the health situation was minimal.

### Measurement instruments

The NMDIP includes 36 items and consists of eight scales and four additional items. The 36 items were divided over the four ICF components. For the Body Functions component items and for the Participation component items scoring options ranged from 0 (no disability) to 4 (complete disability); for the Activities component items scoring options ranged from 0 (no disability) to 3 (complete disability); and for the Environmental Factors component items scoring options ranged from 0 (no support) to 2 (full support) [4]. Item scores were summed into a scale with higher scores indicating more disability. To evaluate the RV, we used the ‘Physical Functioning’ construct as represented by the ‘Activities of Moving around’ and ‘Self-care and Domestic Activities’ scales, the ‘Psychological Functioning’ construct as represented by the ‘Mental Functions and Pain’ scale, and the ‘Social Functioning’ construct as represented by the ‘Participation in Life Situations’ scale. These scales were selected because items in these scales are closely associated with the scales in the concurrent measures.

The SF-36 was selected as a well-known reliable and valid generic multidimensional health-impact measure used for NMD [12, 13]. The SF-36 [8] comprises 36 items with eight functional dimensions. Three scales were used to examine the RV: ‘Physical Functioning’, ‘Mental Health’ and ‘Social Functioning’. Item scores were coded, summed and transformed to a score of 0 (worst health) to 100 (best health) for each scale. The overall Cronbach’s alpha for these scales was 0.79 in a study of Amyotrophic Lateral Sclerosis patients [14]. In our previous study the Cronbach’s alpha for the selected scales ranged from 0.77 to 0.94 [4].

The WHOQOL-bref [9] was selected as a generic measurement instrument for a broad evaluation of quality of life. It consists of 28 items in four constructs and two separate questions. Three scales were used to examine the RV: ‘Physical Health and Autonomy’, ‘Psychological Health’, and ‘Social Relations’. Item scores from each scale were coded, summed and transformed to a score of 0 (worst health) to 20 (best health). The Cronbach’s alpha ranged from 0.63 to 0.81 in a study of Multiple Sclerosis patients [15]. In our previous study the Cronbach’s alpha for the selected scales ranged from 0.60 to 0.84 [4].

The GARS [10] is a domain specific generic measurement instrument for assessing disability in ‘Activities of daily living’ (ADL) and ‘Instrumental activities of daily living’ (IADL). It consists of eleven ADL items and seven IADL items. A four-category response format was used, and ranged from 1 (no problem in performing without help) to 4 (impossible to perform). The scores were summed for each subscale. The Cronbach’s alpha ranged from 0.95 to 0.97 in a study of Multiple Sclerosis patients [15]. In our previous study the Cronbach’s alpha ranged from 0.93 to 0.95 [4].

The IPAQ [11, 16] is a domain specific generic measurement instrument for assessing participation. It consists of fifteen items focusing on person-perceived participation and autonomy. The instrument assesses two aspects of participation: perceived participation and the perceived problems with participation. In this study the perceived participation aspect was used since this construct is closely associated with the ‘Participation in Life Situations’ construct in the NMDIP questionnaire. The sub-domains were ‘Autonomy Indoors’, ‘Family Role’, ‘Autonomy Outdoors’, and ‘Social Relations’. The response options ranged from 1 (very good) to 5 (very poor). Scores were summed for each domain. The Cronbach’s alpha ranged from 0.86 to 0.94 in a study of Multiple Sclerosis patients [15]. In our previous study the Cronbach’s alpha ranged from 0.84 to 0.94 [4].

### Criterion variables

Two questions were selected as criterion variables: ‘Extent of limitations’ and ‘Quality of life’.

To evaluate the ‘Extent of Limitations’ respondents were asked to answer the question: ‘To what extent are you limited due to your NMD?’ Responses were on a ten-point scale ranging from 1 (not limited at all) to 10 (completely limited). Respondents were classified into one of four groups: Group A with a ‘very low extent of limitation’ (score 1–2), Group B with a ‘moderate extent of limitation’ (score 3–5), Group C with a ‘high extent of limitation’ (score 6–8) and, Group D with a ‘very high extent of limitation’ (score 9–10).

The second criterion variable for evaluation of quality of life was one of the two single items adapted from the WHOQOL-bref. Respondents were asked: ‘How would you rate your quality of life?’. Response options were: 1 = very poor, 2 = poor, 3 = neither poor nor good, 4 = good and 5 = very good. Respondents were classified into three groups: Group A– ‘very poor or poor quality of life’, Group B– ‘neither poor nor good’, and Group C– ‘good or very good quality of life’.

### Analysis

Descriptive statistics were used to characterize the total sample and the test-retest sample. Differences between both samples were examined using the difference in proportions test, the two-sample t-test, and if data are not normally distributed a non-parametric test for independent samples were used.

Test-retest reliability or stability over time was examined using the Wilcoxon Signed Test and the one-way random intraclass correlation coefficients (ICCs) [17].

Relative Validity was examined in several steps. First, the Chi-square was computed for each scale by calculating the Kruskal-Wallis H-test. Second, the RV of each scale was computed by dividing each H-statistic by the H-statistic for the scale with the highest H-statistic, and multiplied by one hundred. The resulting RV-estimate indicates the extent to which a scale or construct is able to discriminate between two groups compared to the measure with the highest H-statistic [18, 19]. Finally, the clinical relevance of the differences between respondent subgroups, and the nonparametric effect size (coefficient *r*) for unrelated samples, was calculated for statistically significant group differences (α = 0.05) with post hoc tests (Bonferroni correction) [20]. Effect sizes where estimated through coefficient *r,* which was calculated by dividing the *z*-statistic (obtained from the Mann-Whitney U test) by the root of the sample size (n). To interpret this nonparametric effect sizes (coefficient *r)*, Cohen suggested the following thresholds: an *r* of <0.10 indicates a trivial effect, an *r* of ≥0.10 to <0.24 a small effect, a *r* of ≥0.24 to <0.37 a moderate effect, and an *r* ≥ 0.37 a large effect. A *r* ≥ 0.10 reflects a clinically relevant difference between groups [20, 21].

IBM SPSS statistics version 22 was used.

## Results

A total of 702 participants (70% response rate) completed the questionnaires. Of the 202 patients who agreed to complete the NMDIP twice 185 participants (92% response rate) actually returned the questionnaire.

The non-respondents from the 1003 eligible patients did not differ from respondents in terms of gender, but non-responders were significantly younger than respondents (*p*-value < 0.001 not in table).

The total sample (*n* = 702) and the test-retest sample (*n* = 185) differed in Age, Years since diagnosis. Participants in the total sample were older and were diagnosed more recently with a NMD compared to the test-retest sample. Also a significant larger proportion of respondents in the total sample was ‘Retired due to age’ compared to test-retest sample (*p*-value = 0.007) (Table 1). Finally the NMD category distribution differed significantly between the samples with less patients with Motor-neuron disorders and Muscle disorders and more patients with Peripheral nerve disorders in the total sample compared to the test-retest sample.

**Table 1:** Patient characteristics of total sample and test-retest sample

Variable	Total sample *N* = 702	Test-retest sample *N* = 185	*p*-value
Gender (%)			
Female	350 (50)	105 (57)	0.095^
Male	352 (50)	80 (43)	0.095^
Age			0.024^##^
Median (IQR)	61 (21)	57 (18)	
Range	19–92	19–92	
Year since diagnosis			0.003^##^
Median (IQR)	7 (11)	10 (14)	
Range	0–65	1–64	
Extent of limitations			0.329^##^
Median (IQR)	5 (4)	6 (4)	
Range	1–10	1–10	
Quality of life (WHOQOL-bref)			0.129^##^
Median (IQR)	4 (1)	4 (1)	
Range	1–5	1–5	
Relationship status (%)			
Relationship (married/partnership)	515 (73)	135 (76)	0.910^
Single (unmarried/widowed/divorced)	186 (27)	45 (24)	0.549^
Educational level (%)			
Primary school/vocational training	235 (33)	57 (31)	0.492^
Secondary school/vocational training	270 (38)	81 (44)	0.188^
Higher education /vocational training	161 (23)	37 (20)	0.394^
University	28 (4)	8 (4)	0.837^
Employment status (more answers possible) (%)			
Following a training or study	36 (5)	12 (7)	0.468^
Employment (part-time or full time)	173 (25)	43 (23)	0.693^
Voluntary work (part-time or full time)	42 (6)	15 (8)	0.294^
(Partially) retired due to NMD	213 (30)	67 (36)	0.126^
Housewife/househusband	171 (24)	55 (30)	0.136^
Retired due to age	244 (35)	45 (24)	0.007^
NMD category (%)			
Motor neuron disorder (MND)	43 (6)	20 (11)	0.027^
Muscle disorder (MD)	154 (22)	69 (37)	<0.001^
Junction disorder (JD)	234 (33)	66 (36)	0.549^
Peripheral nerve disorder (PND)	271 (39)	30 (16)	<0.001^

^Difference in proportions test, ^##^Mann-Whitney U test. Interquartile range (IQR) = Q3-Q1

### Test-retest reliability

Wilcoxon Signed Rank Test (Table 2) showed no significant score differences between time points for most of the NMDIP scales, indicating stability over time, except for the ‘Mental Functions and Pain’ scale. However this difference was not clinically relevant (ES 0.18, not shown in table). The ICC of all scales showed sufficient agreement and ranged from 0.79 to 0.97, indicating good stability over time.

**Table 2:** Test-retest reliability for the NMDIP scales (*n* = 185)

	Comparison of scores at measurement 0 and 1						Intraclass correlation (one way random)
	Cases (N)	Median (IQR)	Cases (N)	Median (IQR)	Z-statistic	*p*-value^*^	
		0		1			
Muscle Functions	177	4 (2)	179	4 (2)	−2.08	0.037	0.85
Movement Functions	161	2 (3)	153	2 (2)	−.006	0.995	0.88
Excretion and Reproductive Functions	135	2 (3)	144	1 (3)	−1.00	0.318	0.85
Swallowing and Speech Function	172	0 (2)	180	0 (2)	−0.23	0.818	0.91
Mental Functions and Pain	164	4 (4)	162	4 (3)	−3.39	0.001	0.90
Activities of Moving around	185	4 (6)	185	4 (6)	−1.23	0.219	0.96
Self-care and Domestic Activities	185	3 (9)	185	3 (7)	−0.41	0.683	0.97
Participation in Life Situations	180	1 (2)	182	0 (2)	−1.70	0.090	0.79

^*^Wilcoxon Signed Rank Test, 2-tailed. Interquartile range (IQR) = Q3-Q1

### Criterion-related relative validity

Median scores of patients with a low ‘Extent of limitation’ (Table 3) or very poor or poor ‘Quality of life’ level (Table 4) were significantly different in the hypothesized direction when compared to the next higher group mean.

**Table 3:** Relative validity of the NMDIP, domain specific and generic measurement instruments compared, using subgroups of extent of limitations (*n* = 702)

	Group A (*n* = 110) low extent of limitation		Group B (*n* = 250) moderate extent of limitation		Group C (*n* = 270) high extent of limitations		Group D (*n* = 58) very high extent of limitations		Kruskal-Wallis H		Group A-B^a^	Group B-C^a^	Group C-D^a^	Group A-D^a^
	n	Median (IQR)	n	Median (IQR)	n	Median (IQR)	n	Median (IQR)	Chi Square	RV^b^	es	es	es	es
NMDIP														
Muscle Functions	97	2 (2)	231	3 (2)	263	4 (2)	56	6 (3)	221.3	88	0.44	0.30	0.44	0.78
Movement Functions	92	0 (1)	210	2 (2)	232	3 (2)	49	5 (4)	149.3	59	0.34	0.30	0.29	0.72
Excretion and Reproductive Functions	82	0 (1)	182	1 (2)	194	2 (3)	43	3 (3)	47.1	19	0.16	0.18	-	0.51
Swallowing and Speech Function	107	0 (0)	238	0 (1)	258	0 (1)	55	1 (2)	48.6	19	0.17	0.11	0.18	0.50
Mental Functions and Pain	95	2 (2)	205	4 (3)	237	5 (4)	50	6.5 (5)	128.0	51	0.30	0.29	0.21	0.64
Activities of Moving around	110	0 (2)	250	3 (4)	272	6 (7)	58	13 (9)	251.0	99	0.42	0.39	0.39	0.79
Self-care and Domestic Activities	109	0 (1)	250	2 (3)	272	4 (8)	58	16.5 (14)	219.7	87	0.36	0.35	0.37	0.81
Participation in Life Situations	108	0 (0)	249	0 (1)	271	2 (4)	55	4 (4)	168.5	67	0.24	0.33	0.30	0.76
SF-36														
Physical Functioning	109	27 (6)	250	21 (8)	272	16 (7)	58	11 (3)	252.9	100	0.41	0.38	0.43	0.78
Mental Health	110	25 (3)	250	25 (5)	271	24 (6)	58	21.5 (7)	35.2	14	-	0.12	0.14	0.40
Social Functioning	110	10 (1)	250	8 (2)	271	7 (3)	58	5.5 (4)	129.8	51	0.31	0.26	0.22	0.62
WHOQOL-bref														
Physical Health and Autonomy	108	3 (1)	244	3 (0)	271	3 (0)	57	3 (1)	68.0	27	0.21	0.19	-	0.47
Psychological Health	106	4 (0)	246	3.5 (0.5)	271	3.5 (1)	57	3 (0)	68.0	27	0.19	0.14	0.25	0.55
Social Relations	104	4 (1)	238	4 (0)	261	4 (1)	55	4 (1)	30.8	12	-	0.14	-	0.35
GARS														
Activities of Daily Living	110	11 (1)	250	13 (6)	272	17 (10)	58	27 (17)	213.5	84	0.31	0.35	0.39	0.79
Instrumental Activities of Daily Living	109	7 (2)	250	10 (7)	270	15 (9)	58	24 (5)	215.0	85	0.33	0.35	0.39	0.78
IPAQ														
Autonomy Indoors	110	7 (3)	250	13 (6)	272	14 (6)	57	19 (11)	174.3	69	0.37	0.27	0.32	0.75
Family Role	110	8.5 (7)	248	15 (7)	271	18 (7)	58	22 (9)	177.3	70	0.40	0.28	0.22	0.71
Autonomy Outdoors	110	5.5 (4)	249	9 (3)	272	11 (5)	56	14 (4)	238.5	94	0.39	0.40	0.31	0.76
Social Relations	110	9 (6)	250	12 (4)	272	13 (5)	56	13 (4)	87.0	34	0.24	0.23	-	0.52

nmdip, gars, and ipaq: higher scores = more unable to perform activity; sf-36 and WHOQOL-bref scales: higher scores = better quality of life and more able to perform activity. Interquartile range (IQR) = Q3-Q1. - = not statistically significant. Bonferroni correction^a^ = 0.0125 (*p*-value 0.05/4). RV = relative validity,^b^ = score indicating the relative validity with score 100 related to the highest H-statistic*.* ES = effect size

**Table 4:** Relative validity of the NMDIP, domain specific and generic measurement instruments compared, using subgroups of Quality of Life (*n* = 702)

	Group A (*n* = 53) Very poor or poor QoL		Group B (*n* = 175) Neither poor nor good QoL		Group C (*n* = 474) Good or very good QoL		Kruskal-Wallis H Chi Square	RV^b^	Group A-B^a^	Group B-C^a^	Group A-C^a^
	n	Median (IQR)	n	Median (IQR)	n	Median (IQR)			es	es	es
NMDIP											
Muscle Functions	52	5 (2)	167	4 (2)	439	3 (2)	81.4	50	0.34	0.23	0.35
Movement Functions	46	4 (3)	148	3 (2)	400	2 (3)	83.8	51	0.30	0.27	0.35
Excretion and Reproductive Functions	36	3 (5)	129	2 (2)	344	1 (2)	40.6	25	0.21	0.22	0.25
Swallowing and Speech Function	49	2 (3)	162	0 (1)	458	0 (1)	43.6	27	0.26	0.14	0.27
Mental Functions and Pain	45	8 (4)	147	6 (4)	405	3 (4)	147.7	90	0.28	0.42	0.40
Activities of Moving around	53	10 (8)	175	4 (6)	474	3 (5)	75.2	46	0.36	0.19	0.35
Self-care and Domestic Activities	53	10 (13)	175	3 (8)	473	2 (4)	79.9	49	0.32	0.22	0.33
Participation in Life Situations	52	4.5 (5)	175	2 (4)	468	0 (2)	103.7	63	0.32	0.26	0.39
SF-36											
Physical Functioning	53	12 (4)	175	17 (8)	473	21 (10)	85.7	52	0.38	0.23	0.34
Mental Health	53	20 (6)	175	22 (6)	473	25 (4)	132.5	81	0.27	0.35	0.38
Social Functioning	53	5 (3)	175	7 (2)	473	9 (2)	138.3	84	0.31	0.36	0.38
WHOQOL-bref											
Physical Health and Autonomy	53	3 (5)	173	3 (0)	466	3 (0)	117.7	72	0.21	0.41	0.34
Psychological Health	53	3 (0)	173	3 (1)	466	4 (0)	163.8	100	0.31	0.40	0.41
Social Relations	50	3 (1)	170	4 (1)	450	4 (0)	61.3	37	-	0.25	0.25
GARS											
Activities of Daily Living	53	26 (16)	175	17 (10)	474	13 (7)	80.6	49	0.37	0.20	0.35
Instrumental Activities of Daily Living	52	22 (12)	175	15 (9)	472	11 (9)	82.8	51	0.34	0.23	0.33
IPAQ											
Autonomy Indoors	53	19 (9)	175	14 (5)	473	12 (7)	109.6	67	0.32	0.29	0.36
Family Role	53	24 (8)	175	20 (8)	471	15 (8)	127.6	78	0.28	0.34	0.37
Autonomy Outdoors	53	15 (4)	174	11 (5)	471	8 (5)	157.6	96	0.42	0.35	0.43
Social Relations	53	15 (6)	175	13 (3)	472	11 (5)	134.1	82	0.26	0.35	0.37

nmdip, gars, IPAQ scales: higher scores = lower quality of life, sf-36, and WHOQOL-bref scales: higher scores = higher quality of life. Interquartile range (IQR) = Q3-Q1. - = not statistically significant. Bonferroni correction^a^ = 0.02 (*p*-value 0.05/3). *RV* relative validity.^b^ = score indicating the relative validity with score 100 related to the highest H-statistic. *ES* effect size

### Extent of limitations

About 16% (*n* = 110) of the respondents reported ‘low extent of limitations’ (Group A) due to NMD, while 36% (*n* = 250) reported a ‘moderate extent of limitation’ (Group B), and 39% (*n* = 270) reported a ‘high extent of limitation’ (Group C). About 8% (*n* = 58) of the respondents reported a ‘very high extent of limitations’ (Group D).

Comparisons of the RV coefficients, as summarized in Table 3, revealed that the NMDIP ‘Activities of Moving around’ scale and SF-36 ‘Physical Functioning’ scale were the most valid in discriminating between groups with an increasing extent of limitation.

We then examined the performance of the NMDIP-scales in indicating the differences between extreme groups (A-D) and subgroups (A-B, B-C, C-D) regarding the physical-, psychological- and social functioning constructs, as they relate to similar constructs in the concurrent measurement instruments. Regarding physical functioning, we found that both NMDIP activity scales turned out to be the most sensitive (followed by the ‘Muscle Functions’ scale) for measuring differences between extreme groups and subgroups. However, the performance of the concurrent SF-36 ‘Physical functioning’ scale and both GARS scales were almost identical. Regarding the psychological functioning construct we found that the NMDIP ‘Mental Functions and Pain’ scale was the best performing scale compared to the SF-36 ‘Mental Health’ scale and the WHOQOL-bref ‘Psychological Health’ scale, showing the highest extreme group and subgroup differences. Regarding the social functioning construct the NMDIP ‘Participation in Life Situations’ scale performed better than the SF-36 ‘Social Functioning’ and the WHOQOL-bref ‘Social Relations’ scales, and roughly as well as the same as the comparable constructs in the domain-specific IPAQ.

In summary, the NMDIP scales performed sufficient to good in discriminating between (sub) groups with an increasing extent of limitations compared to similar constructs in concurrent measures regarding physical functioning, psychological functioning and social functioning constructs.

### Quality of life

Eight percent (*n* = 53) of the respondents reported poor or very poor quality of life (Group A), while 25% (*n* = 175) experienced their quality of life as neither poor nor good (Group B) and 67% (*n* = 474) reported a good or very good quality of life’ (Group C).

Comparisons of the RV-coefficients, as summarized in Table 4, revealed that the SF-36 ‘Psychological Health’ scale and IPAQ ‘Autonomy outdoors’ scales were the most valid in discriminating between groups with differences in quality of life. The ‘Mental Functions and Pain’ NMDIP scale was the third most valid scale.

When examining the performance of the NMDIP-scales in indicating the differences between extreme and subgroups for quality of life, we found about the same extreme group differences for the physical functioning scales for all concurrent constructs with moderate Effect Sizes (ESs). The same goes for the subgroup differences, although the NMDIP ‘Mental Functions and Pain’ scale, and the WHOQOL-bref ‘Psychological Health’ scale performed slightly better than the SF-36 ‘Mental Health’ scale. Finally, when examining the social functioning scales we found that the comparable NMDIP ‘Participation in Life Situations’ scale performed about as well as the SF-36 ‘Social Functioning’ scale and the IPAQ scales with a moderate to large ESs for extreme group differences. The NMDIP ‘Participation in Life Situations’ scale also performed better compared to the social functioning construct of the WHOQOL-bref, the ‘Social Relations’ scale. The same goes for the subgroup differences.

In summary, the NMDIP scales performed well in discriminating between subgroups with differences in quality of life compared to similar constructs in concurrent measures concerning the physical functioning, psychological functioning and social functioning constructs.

## Discussion

In this study the NMDIP, that was developed to reflect the prevalence and severity of a broad range of NMD-related disabilities [4], showed stability and performed well in the criterion-related subgroups of NMD-patients who differed in the extent of limitation and quality of life.

The results of the test-retest reliability analysis were sufficient indicating stability in the eight NMDIP scales. Although the results showed a difference for ‘Mental Functions and Pain’ scale while the effect size was trivial, the intraclass correlation showed sufficient agreement for all NMDIP scales between the two measurement moments.

In general, the NMDIP scales performed well in discriminating between relevant subgroups with increasing extent of limitation. This was the case for constructs evaluating physical, psychological, and social functioning. The NMDIP scales showed satisfactory relative validity and moderate to strong ESs indicating the strength of the differences between subgroups. The NMDIP showed satisfactory performance in discriminating between relevant subgroups with decreasing Quality of Life. This was the case for constructs evaluating physical, psychological and social functioning.

Strength of this study is the inclusion of a large population of patients diagnosed with a NMD. Some potential study limitations should be mentioned. First, RV was examined as criterion-related validity value in this study. Because of the absence of a widely accepted criterion measure we chose to use self-report measures, which turned out to be a useful method. Secondly, the (relatively) small group sizes for ‘very high extent of limitations’ (Group D) and ‘very poor or poor quality of life’ (Group A) might have a negative impact on detecting group differences, though the difference between these subgroups and the adjacent groups showed sufficient ESs.

The results in this study permit us to recommend that researchers consider Relative Validity as a useful method to select a valid and ‘with caution’ a sensitive measure, especially when data from longitudinal studies or intervention studies are lacking. At the same time, we want to stress that RV is not a substitute for the sensitivity-to-change test. The findings in this study cannot be generalized to longitudinal studies. We recommend further research to evaluate the sensitivity to change of the NMDIP scales.

Furthermore generic health measures have some disadvantages against disease-specific health measures in addressing topics of a particular relevance to patients with a specific disease. Therefore it is recommended that the individual items in a scale be examined to estimate the suitability of the scale for a particular patient population [13].

## Conclusions

The results in this study confirmed the stability of the NMDIP over time, and showed good relative validity compared to generic QOL and domain-specific measures. In combination with the findings in our previous study [4], the NMDIP proved to be a valid and reliable disease-targeted measure with a broad scope on physical, psychological and social functioning. Further research should examine the responsiveness of the NMDIP scales.

## Abbreviations

ADL: Activities of daily living
GARS: Groningen Activity Restriction Scale
IADL: Instrumental activities of daily living
ICC: Intraclass correlation coefficient
IPAQ: Impact on Participation and Autonomy Questionnaire
NMD: Neuromuscular Diseases
NMDIP: Neuromuscular Disease Impact Profile
RV: relative validity
SF-36: Medical Outcome study Short Form Questionnaire
WHOQOL-bref: World Health Organization Quality Of Life-abbreviation version
